# Medical Education Partnership Initiative gives birth to AFREhealth

**DOI:** 10.1016/S2214-109X(17)30329-7

**Published:** 2017-10

**Authors:** Francis Omaswa, Elsie Kiguli-Malwadde, Peter Donkor, James Hakim, Milliard Derbew, Sarah Baird, Seble Frehywot, Onesmus Wairumbi Gachuno, Steve Kamiza, Isaac Ongubo Kibwage, Kein Alfred Mteta, Yakub Mulla, Fitzhugh Mullan, Jean B Nachega, Oathokwa Nkomazana, Emilia Noormohamed, Vincent Ojoome, David Olalaye, Sandy Pillay, Nelson K Sewankambo, Marietjie de Villiers

**Affiliations:** African Centre for Global Health and Social Transformation, 13B Acacia Avenue, Kampala 9974, Uganda (FO, EK-M, VO); Kwame Nkrumah University of Science and Technology, Kumasi, Ghana (PD); University of Zimbabwe, Harare, Zimbabwe (JH); Addis Ababa University, Addis Ababa, Ethiopia (MD); George Washington University, Washington, DC, USA (SB, SF, FM); University of Nairobi, Nairobi, Kenya (OWG, IOK); University of Malawi, College of Malawi, Chichiri, Malawi (SK); Kilimanjaro Christian Medical University College, Moshi Tanzania (KAM); University of Zambia, Lusaka, Zambia YM); Stellenbosch University, Stellenbosch, South Africa (JBN, MdV); University of Botswana, Gaberone, Botswana (ON); Universidade Eduardo Mondlane, Maputo, Mozambique (EN); University of Ibadan, Ibadan, Nigeria (DO); University of KwaZulu-Natal, Durban, South Africa (SP); and Makerere University, Kampala, Uganda (NKS)

The African Forum for Research and Education in Health (AFREhealth) held its first symposium in Accra, Ghana, between Aug 1 and Aug 3, 2017. This new organisation is committed to developing health professionals’ education and research in Africa, sharing best practices, and reducing health disparities. AFREhealth aims to partner with stakeholders to improve health outcomes, work towards an AIDS-free generation, establish a research agenda for health priorities in Africa, and mobilise vital resources. It is an outcome of the Medical Education Partnership Initiative (MEPI) and the Nursing Education Partnership Initiative. Here we highlight how the achievements of MEPI have inspired the formation of AFREhealth.

MEPI was a US$130 million competitively awarded grant by the US President’s Emergency Plan for AIDS Relief and National Institutes of Health to 13 medical schools in 12 sub-Saharan African countries and a Coordinating Center at George Washington University, DC, USA. Implementation was led by principal investigators from the grantee institutions, supported by the Health Resources and Services Administration, National Institutes of Health, and the Coordinating Center, between September, 2010 and August, 2015. Each grantee worked with partner institutions in the USA, Europe, and Africa. The goals were to increase the capacity of the awardees to produce more and better trained doctors, strengthen locally relevant research, promote retention of graduates within their countries, and ensure sustainability of the improvements that the project supported. MEPI implementation was overseen by the Principal Investigators Council, which included principal investigators from grantee institutions, and representatives from the US government and the Coordinating Center. Unlike some other donor grants, MEPI grants were awarded directly to the principal investigator at the African institution, who then determined the direction of their programmes within the context of the Request for Application.

MEPI generated excitement and hope among the grantee schools and countries, and recorded the following key achievements. First, the African principal investigators expanded MEPI from 13 grantees to over 60 medical schools in Africa by creating in-country consortia that shared resources and experience. This expansion helped to create strong South-to-South and South-to-North partnerships in medical education and research. To date, MEPI has led to the establishment of ten new schools, doubled student intake in some schools, increased postgraduate student numbers threefold, and improved faculty expansion and retention.^1^ Second, grantees changed curricular to competency-based models that were more responsive to the health priorities of each country and ensured better delivery of such curricular by creating Medical Education Units to improve the quality of teaching and learning. All schools embraced e-Learning by enhancing infrastructure, improving internet connectivity (including at off-campus satellite training sites), installing more computers, and restructuring library spaces to facilitate e-Learning. The number of students with personal internet-enabled devices increased from 5% to 90%.^1^ Third, research support centres were established to promote institutional and collaborative research. These centres facilitated training in research methodology, grant writing, scientific writing, and research administration, which help to build a sustainable research environment. These factors improved the schools’ ability to attract funding, recruit and retain faculty, and contribute to the national research agenda and sustainability of the programmes. Fourth, joint learning was facilitated through site visits to grantees for peer learning and management support; annual symposia showcased work through scientific papers, posters, and networking; eight content-based Technical Working Groups held face-to-face meetings and monthly virtual meetings on topics such as community-based education, competency-based education, monitoring and evaluation, graduate tracking, library and information sciences, medical education research, and research support centres. Over 376 peer-reviewed publications, including a special supplement in *Academic Medicine* in 2014,^2^ communicated the research findings and medical-education-related successes of MEPI. Fifth, the increase in the number of students intending to remain and work in one of the countries (5% to 80% over the study period) and the creation of new postgraduate courses were early evidence of the successful retention of graduates. For example, the University of Zambia introduced 14 new postgraduate courses—mainly in basic sciences—with an intake of 70 students.^2^ It is too early to tell if MEPI has affected health systems.

Looking ahead, high-quality health professionals are needed in Africa to ensure global health security and achievement of African and global health goals. The international community is interested in what AFREhealth can achieve, given MEPI has demonstrated availability of opportunities for collaboration and joint learning in Africa. Sustained MEPI achievements depend on African leadership, continued stability, and economic growth in African countries. These conditions are needed for to create an environment and fiscal space to employ and retain high-calibre faculty and graduates, support research, and maintain educational infrastructure. The first AFREhealth symposium was therefore greatly anticipated, and was a resounding success.
